# Mesenchymal stem cells under epigenetic control – the role of epigenetic machinery in fate decision and functional properties

**DOI:** 10.1038/s41419-023-06239-4

**Published:** 2023-11-06

**Authors:** Alicja Walewska, Adrian Janucik, Marlena Tynecka, Marcin Moniuszko, Andrzej Eljaszewicz

**Affiliations:** 1 Centre of Regenerative Medicine, Medical University of Bialystok, ul. Waszyngtona 15B, 15-269 Bialystok, Poland; 2 Department of Regenerative Medicine and Immune Regulation, Medical University of Bialystok, ul. Waszyngtona 13, 15-269 Bialystok, Poland; 3 Department of Allergology and Internal Medicine, Medical University of Bialystok, ul. M. Sklodowskiej-Curie 24A, 15-276 Bialystok, Poland; 4 Tissue and Cell Bank, Medical University of Bialystok Clinical Hospital, ul. Waszyngtona 13, 15-069 Bialystok, Poland

**Keywords:** Mesenchymal stem cells, DNA methylation, Stem-cell differentiation, Epigenetic memory, Stem-cell research

## Abstract

Mesenchymal stem cells (mesenchymal stromal cells, MSC) are multipotent stem cells that can differentiate into cells of at least three mesodermal lineages, namely adipocytes, osteoblasts, and chondrocytes, and have potent immunomodulatory properties. Epigenetic modifications are critical regulators of gene expression and cellular differentiation of mesenchymal stem cells (MSCs). Epigenetic machinery controls MSC differentiation through direct modifications to DNA and histones. Understanding the role of epigenetic machinery in MSC is crucial for the development of effective cell-based therapies for degenerative and inflammatory diseases. In this review, we summarize the current understanding of the role of epigenetic control of MSC differentiation and immunomodulatory properties.

## Facts

- Major transcription factors determining MSCs’ are epigenetically controlled.
- C/EBPα (encoded by CBPA) and PPARγ (encoded by PPARG) are recognized as master regulators of adipocyte differentiation. The expression of *CEBPA* depends on HDAC1 activity. Opening of PPARG promoter region depends on SWI/SNF complex and simultaneous acetylation of histone H3 residues.
- RUNX2 and OSX major orchestrators of osteogenic differentiation are controlled by, methylation at histone H3 residues, acetylation at histone H3 and H4 residues, and DNA methylation signatures.
- Chondrogenic differentiation depends on MSCs condensation to form 3D structures. SOX9 is a major transcription factor in early chondrogenesis, and its expression is controlled by histone H3/H4 acetylation.
- MSCs licensing with proinflammatory cytokines, hypoxia conditions, and bioactive molecules induces their immune-modulatory activities.

## Open questions

- Do differentially sourced MSCs differ in the epigenetic landscape, showing higher environmentally defined potential for differentiation or immune modulation (trained effect)?
- What is the role of ncRNAs in the regulation of epigenetic machinery in MSCs?
- What are the epigenetic mechanisms controlling MSCs immunomodulatory properties after licensing with proinflammatory cytokines?
- How stress microenvironment is changing the epigenetic landscape of MSCs?
- How to modulate MSCs epigenetic landscape to improve their therapeutic properties?

## Introduction

Mesenchymal stem cells (MSCs, also known as mesenchymal stromal cells and multipotent stromal cells) were first identified in the bone marrow by Alexander Friedenstein et al. in the late '60s [1, 2]. They were described by Owen et al. as spindle-shaped, colony-forming unit fibroblasts (CFU-Fs) [3]. Nowadays, MSCs are successfully isolated and characterized from almost every tissue, including adipose tissue, lungs, liver, kidney, peripheral blood, umbilical cord, Wharton’s jelly, and dental pulp [4, 5]. To date, several phenotypical and functional variances among differentially sourced MSCs have been reported, which started a scientific debate over the homogeneity and their therapeutic properties. In 2005, the International Society for Cellular Therapy proposed three minimal criteria to define human MSCs, namely: i) plastic or glass surface adherence; ii) the ability to differentiate into at least adipogenic, chondrogenic, and osteogenic lineages in vitro; and iii) the presence of specific phenotype associated with the expression of CD29, CD90, CD73 with simultaneous lack of CD14, CD34, CD45, and HLA-DR expression [6–8].

MSCs gained considerable interest due to their promising therapeutic potential in degenerative and inflammatory diseases. The lack of immunogenicity offers the possibilities of their applications both in autologous and allogeneic systems [9]. Notably, MSCs activities such as self-renewal, differentiation, and immunoregulation can be modulated by microenvironmental factors. Consequently, MSCs’ stability remains the major safety issue, limiting their broad use in clinical settings. Therefore, a better understanding of MSCs’ biology and the effects of microenvironmental stimulation on their function is crucial to approximate their therapeutical usage. Notably, our understanding of the role of epigenetic machinery in cellular biology allowed us to define complex molecular mechanisms controlling cell phenotype and functional properties. It seems that, similarly to other stem and adult cells, epigenetic modifications play an important role in MSCs’ fate decisions and functions. Here, we aimed to summarize current progress in understanding the role of epigenetic mechanisms controlling MSCs’ differentiation and immunomodulatory function.

## The mechanism of epigenetic modifications

All cellular processes and functions depend on the expression of genes encoding proteins or regulatory molecules. In fact, gene expression is a fundamental process that enables the decoding of the DNA sequences into the final functional gene product (cellular proteins). Notably, DNA is tightly packed within the nucleus of eukaryotic cells in highly organized and compact structures, making the gene hardly accessible to transcription machinery. Nucleosomes represent the first level of DNA packing, formed by DNA wrapped around histone proteins. Histones are nucleosome core proteins providing structural support to form chromatin. Five types of histones were identified. The nucleosome is formed by core histones, namely H2A, H2B, H3, and H4, while H1 and H5 are involved in higher-order chromatin structures [10]. Gene expression depends on chromatin accessibility, defined as the extent of physical interaction between nuclear macromolecules and “chromatinized” DNA. The structure of chromatin may be lightly packed (referred to as euchromatin) or tightly packed - more condensed (referred to as heterochromatin). Chromatin accessibility is controlled by the occupancy and topological arrangement of nucleosomes, along with the presence of various chromatin-binding factors, which bind either directly or indirectly to DNA, that modulate DNA accessibility to transcription machinery (extensively reviewed in ref. [11]). Progress in understanding molecular mechanisms regulating gene expression allowed us to define epigenetics.

**Fig. 1:**
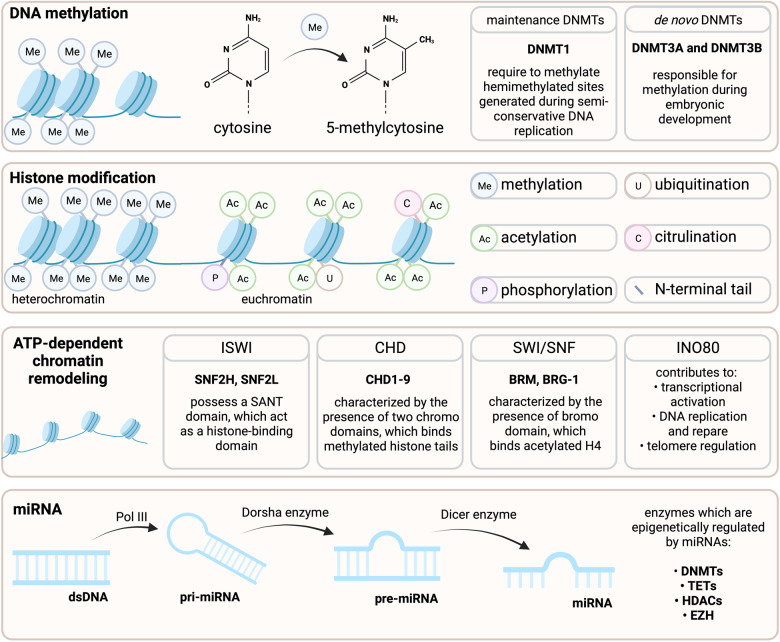
DNA methylation, histone modifications, ATP-dependent chromatin remodeling, and miRNAs mediate epigenetic changes. Chromatin is composed of proteins (histones) and DNA that form chromosomes, and the spatial organization of chromatin is critical for gene expression regulation. Epigenetic machinery, including methylation of DNA and histone modifications, directly orchestrates chromatin activity, mediating the balance between an inactive (heterochromatin) or an active form (euchromatin). DNA methylation occurs by adding a methyl group (-CH3) to cytosine. This process is catalyzed by enzymes such as the activity of DNA methyltransferases (DNMTs). Histone modification includes methylation (Me), acetylation (Ac), phosphorylation (P), ubiquitination (U), and citrullination (C) on N-terminal tails. ISWI, CHD, SWI/SNF, and INO80 are the major subfamilies of ATP-dependent chromatin remodelers. In contrast to above mentioned epigenetic modifications, miRNAs are not directly involved in chromatin activity; however, as potent posttranslational regulators of gene expression, miRNAs may regulate the expression of enzymes involved in direct modifications of chromatin, including DNMTs, ten-eleven translocation (TETs) methyl dioxygenases (involved in DNA demethylation), histone deacetylases (HDACs), and histone-lysine N-methyltransferase enzymes belonging to EZH family (enhancer of zest homolog). Created with BioRender.com.

Epigenetics refers to the heritable changes in gene expression and long-term alteration in the transcriptional potential at the chromatin level [12] (Fig. 1). The first evidence was reported by Waddington C.H. et al. in 1942 and defined as a phenomenon above genetics [13]. Currently, we understand that epigenetic regulation of gene expression may be mediated by DNA methylation, histone modifications, and chromatin remodeling, which occur without changes in the DNA sequences. In addition to the above-mentioned mechanisms associated with direct chromatin remodeling, control of gene expression by regulatory non-coding RNAs (ncRNAs) is usually considered a part of the epigenetic machinery. In this chapter, we will briefly introduce the essential epigenetic mechanisms associated with direct chromatin modifications that will be further discussed in the context of MSCs’ fate and immunomodulatory function.

### DNA methylation

DNA methylation is a biological process involving direct chemical modification of the DNA without changing its sequence [14]. This process is controlled by catalytic enzymes belonging to the family of methyltransferases (DNMTs), transferring a methyl group (-CH_3_) from S-adenosylmethionine (AdoMet/SAM) to cytosine in a dinucleotide sequence CpG (extensively reviewed in ref. [15]). Approximately 70% of gene promoter sites are associated with CpG islands (CGIs) which are responsible for transcriptional initiation [16]. The canonical DNMT enzymes, namely DNMT3a and DNMT3b, can establish de novo methylation to unmodified sequences [17]. At the same time, DNMT1 maintains the methyl group transfer from parental to newly synthesized DNA strand during DNA replication [18, 19]. DNA methylation was shown to play a crucial role in genomic imprinting, regulation of tissue-specific gene expression, and X chromosome inactivation; therefore, it is recognized as a conservative and long-lasting mechanism [20, 21].

### Histone modifications

Chromatin structure is dense and hardly accessible for enzymes involved in transcription processes. Therefore, histones undergo post-transcriptional modifications to control DNA accessibility. To date, four mechanisms of histone modifications are best described and functionally characterized, namely acetylation, methylation, ubiquitination, and phosphorylation [22].

Histone acetylation is controlled by acetyltransferases (HATs) that transfer acetyl groups from acetyl co-enzyme A to lysine residues of the N-terminal H3 and H4 tails [23, 24]. Consequently, the positive charge of histone is neutralized, which reduces chromatin condensation, allowing the initialization of gene transcription [25]. This process may be reversed by activating histone deacetylases (HDACs), which remove acetyl groups from lysine residues making chromatin denser and more unavailable for transcriptional machinery and thus repressing gene expression [26, 27].

Similarly, to DNA methylation, methyl groups can be transferred to amino acids of histone proteins by methyltransferases; however, this process is independent of DNA methylation. Histone methylations can both repress or activate transcriptional processes depending on the methylation region, and a number of modifications occurred [28]. Histone methylation may occur on all histones, targeting especially lysine and arginine residues [29].

Ubiquitination refers to modifying the epsilon amino group of lysine by attaching one (monoubiquitinylation) or more (polyubiquitination) ubiquitin monomers. Ubiquitin is a small protein found in the vast majority of human tissues. Similarly, to the processes mentioned above, ubiquitination occurs in lysine residues of the N-terminal histone tails. Histones H2A and H2B seem to be the most ubiquitinated proteins in the nucleus [30, 31]. However, some reports showed that H1, H3, and H4 could also be modified by ubiquitination [32]. The process is controlled by several different enzymes such as Ubiquitin C-termin Hydrolase 1 (UCHL1), and Ubiquitin Specific Peptidase 1 (USP1) (for an extensive review, please see ref. [33]) and may control transcription processes, DNA damage responses, stem cell maintenance, and differentiation, among others [34]. However, it seems that H2A monoubiquitinylation is frequently associated with the repression of gene transcription [35, 36], while H2B monoubiquitinylation is often associated with increased transcription [37–39].

Contrary to the abovementioned processes, histone phosphorylation occurs on serine, thyronine, and threonine. Their role in the regulation of transcription remains not fully elucidated, although a number of proteins with phosphor-binding domains recognizing phosphorylated histones have been characterized [40, 41]. It seems that histone phosphorylation is primarily associated with the response to DNA damage (for review, please see ref. [42]).

### Adenosine triphosphate-dependent chromatin remodeling

Adenosine triphosphate (ATP)-dependent chromatin alternations are mediated by multi-subunit chromatin-remodeling complexes utilizing the energy from ATP hydrolysis. These ATP-dependent chromatin remodeling enzymes were classified within the RNA/DNA helicase 2 superfamily and, based on their ATPase domain sequence similarities, may be divided into four groups/subfamilies, namely chromodomain helicase DNA-binding (CHD), SWItch/Sucrose non-fermentable (SWI/SNF), imitation SWI/SNF (ISWI), and INO80 (reviewed extensively in ref. [43]). Their direct role in developmental and physiological processes is well established. Interestingly, they may also be recruited by tissue-specific transcription factors to gene promoters and interact with other epigenetic modifiers such as HATS, HDACs, and histone methyltransferases (HMTs) [44].

### Non-coding RNAs

ncRNAs are widely present in the eukaryotic genome and in contrast to messenger RNA do not translate into proteins. They are classified into housekeeping or regulatory ncRNAs. Housekeeping ncRNAs include ribosomal RNA (rRNA), transfer RNA (tRNA), small nuclear RNA (snRNA), small nucleolar RNA (snoRNA), and telomerase RNA (TERC). They are involved in fundamental cellular processes, including mRNA translation (rRNA, tRNA), pre-mRNA splicing (snRNA), RNA modifications (snoRNA), and telomeric DNA synthesis (TERC). In contrast, regulatory ncRNAs are considered functional components of gene expression, which are usually classified into two subclasses, namely small ncRNAs including microRNA (miRNA; 18-22 nt), small interfering RNA (siRNA; 20-25 nt), piwi RNA (piRNA; 26-31 nt), small cajal body-specific RNA (scaRNA; 200-300 nt), and long ncRNAs including long intergenic ncRNA (lincRNA; 1 kb nt), circular RNA (circRNA; 100-999 nt), and natural antisense transcript (NAT; >200 nt). The importance and function of ncRNAs in cellular biology were extensively reviewed in ref. [45]. In fact, regulatory ncRNAs play a crucial role in almost all cellular processes, regulating gene expression at the posttranscriptional level [46, 47]. However, ncRNAs do not directly modify DNA or chromatin structure. Although they can indirectly influence epigenetic modifications, such as recruiting histone modifiers or regulating the expression of proteins and enzymes involved in this process, they do not directly modify the epigenetic landscape of the genome [48–50]. Therefore, in this review, we will summarize the function of ncRNAs directly regulating the expression and activation of molecules involved in the epigenetic modifications (Table 1).

**Table 1:** The involvement of non-coding RNAs into epigenetic regulation of mesenchymal stem cell differentiation.

Non-coding RNA	Mechanism	Source	Model	Refs.
miR-143	Regulate adipogenesis through MAP2K5 pathway	hAD-MSC	In vitro	[54]
lncRNA TINCR	Upregulate adipogenesis by sponging miR-31-5p in a lncRNA TINCR/miR-31-5p/C/EBPα feedback loop	hAD-MSC	In vitro	[59]
miR-675	Inhibit adipogenesis through targeting 3’UTR of HDAC4-6	hBM-MSC	In vitro	[67]
miR-130a, and miR-27b	Promote osteogenesis through inhibitory effect on PPARγ	hBM-MSC	In vitro	[77]
lncRNA TCONS_00041960	Acting as a sponge for Runx2 targeting miR-204-5p, miR-608-5p, and miR-30b-3p respectively	rat BM-MSC	In vitro	[88]
lncRNA TCONS_00023297		hBM-MSC		[89]
lncRNA CALB2		hDPSCs		[90]
miR-145	Inhibits chondrogenic differentiation by directly targeting to the Sox9 3’UTR	hBM-MSC	In vitro	[116]
miR-495				[117]
miR-574-3p	Regulate chondrogenesis through RXRα target	hBM-MSC	In vitro	[118]

*miRNA* micro RNA, *hAD-MSC* human adipose tissue-derived mesenchymal stem cells, *hBM-MSC* human bone marrow-derived mesenchymal stem cells, *lncRNA* long non coding RNA, *HDAC* histone deacetylase, *MAP2K5* Mitogen-Activated Protein Kinase Kinase 5, *PPARγ* Peroxisome Proliferator Activated Receptor Gamma, *Sox9* - SRY-box transcription factor 9, *RXRα* Retinoid X Receptor Alpha.

## Epigenetic control of mesenchymal stem cell differentiation

As mentioned, MSCs present the plasticity to differentiate into mesodermal lineage cells, namely adipocytes, osteocytes, and chondrocytes. Nowadays, it has become clear that epigenetic reprogramming in response to the mediators regulating MSCs’ fate (growth factors, cytokines, and metabolites) plays a vital role during differentiation. Here we will summarize our understanding of epigenetic control of MSCs differentiation.

### Adipogenic differentiation

**Fig. 2:**
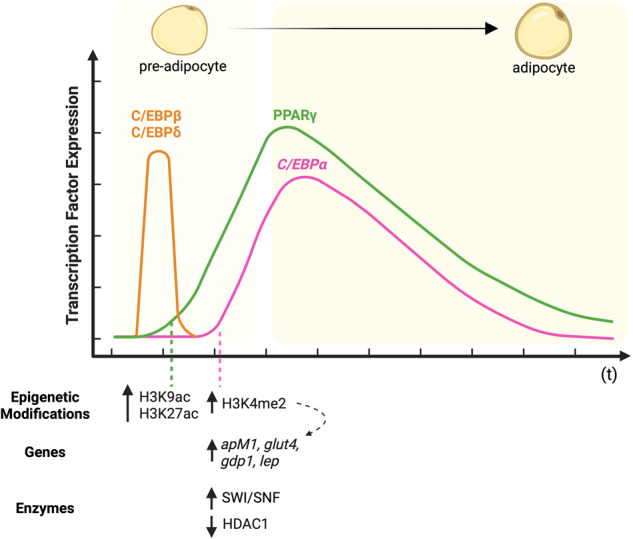
Epigenetic changes in the course of adipogenic differentiation. Adipogenesis occurs in a two-step process, namely the determination phase, followed by the differentiation phase. Adipogenic differentiation induction attracts CCAAT/enhancer-binding proteins beta and delta (C/EBPβ and C/EBPδ, respectively) activation and binding to peroxisome proliferator-activated receptor gamma (*PPARG*) promoter encoding PPARγ Simultaneously HDAC1 activity is decreased, which in turn activates the transcription of C/EBPα. C/EBPα and PPARγ are the major transcription factors whose expression is controlled by the acetylation (ac) of histone H3 lysine 9 and 27 (H3K9ac mark and H3K27ac mark, respectively). Further, di-methylation of histone H3 lysine 4 (H3K4me2 mark) upregulates *adiponectin (apM1), glucose transporter type 4 (glut4), g-patch domain protein 1 (gdp1)*, and *leptin (lep)* gene expression. Created with BioRender.com.

The adipogenic differentiation of MSCs required changes in the epigenetic landscape, allowing activation of the specific transcription machinery inducing cellular plasticity and further adipocyte maturation. Differentiation toward adipocytes is usually considered a two-step process, namely determination (described as a commitment to the adipocyte lineage) followed by differentiation (adipogenesis) (Fig. 2).

The CCAAT/enhancer-binding protein alpha (C/EBPα encoded by *CEBPA*) and the peroxisome proliferator-activated receptor gamma (PPARγ, encoded by *PPARG*) are recognized as master regulators of adipocyte differentiation that control both stages of the process. The *PPARG* expression is activated after a few hours of differentiation induction by CCAAT/enhancer-binding proteins beta and delta (C/EBPβ and C/EBPδ, respectively) binding to the *PPARG2* promoter [51–53]. In addition, PPARγ phosphorylation is reduced by miR-143 mediated downregulation of dual specificity mitogen-activated protein kinase kinase 5 (MAP2K5) expression and subsequent blocking of MAP2K5–ERK5 (extracellular signal-regulated kinase 5) signaling pathway [54].

From the perspective of the epigenetic machinery, the opening of the *PPARG* promoter region seems to depend on the activity of the SWI/SNF complex and is accompanied by acetylation of histone H3, namely acetylation of the 9^th^ lysine (K9ac) residue of histone H3 (described as H3K9ac mark) and H3K27ac mark [55]. SWI/SNF complex activity has been shown to be regulated by lncRNAs such as SWINGN [56]. However, to date, it remains elusive whether this mechanism may be involved in the regulation of adipogenic differentiation of MSCs. The expression of *CEBPA* gene is initially blocked by HDAC1 activity, which is further downregulated upon accumulation of PPARγ through 26 S proteasome degradation, allowing C/EBPα expression [57, 58]. Moreover, C/EBPα activity promotes the expression of lncRNA TINCR, which acts as a sponge to reduce miR-31-5p and, in turn, unblock C/EBPα overexpression [59]. C/EBPα controls the expression of downstream genes by recruiting SWI/SNF complex to the promoter region of its target genes [60].

Committed cells remain undifferentiated and possess the phenotypic characteristic of MSCs. During the determination step, DNA demethylation in the promoter regions of genes critical for cell fate decision occurs. This process is accompanied by di-methylation of the 4^th^ lysine (K4me2) residue of histone H3 (described as H3K4me2 mark) in the promoter regions of late adipogenic genes such as *adiponectin* (*apM1), glucose transporter type 4 (glut4), g-patch domain protein 1 (gdp1)*, and *leptin* (*lep)* [61]. Interestingly, however, this activation mark is not associated with the active transcription of those genes in preadipocytes. Further, during the differentiation step, their active transcription is associated with DNA demethylation of their promoters, with simultaneous histone demethylation and histone H3 acetylation [62, 63]. Histone H3 acetylation seems to be mediated by CREB-binding protein (CBP), p300HAT, and simultaneous reduction of HDAC1 and HDAC3 activity [64, 65]. The process may be controlled by Sirt 1 (NAD-dependent) and HDAC3 (Zn-dependent) deacetylases to inhibit adipogenesis by decreasing *PPARG* expression [66]. In addition, upregulation of HDAC4-6 activity was observed during adipogenic differentiation of bone marrow-delivered MSCs [67]. Despite an increasing body of functional evidence indicating the significant importance of class II HDACs in the adipogenic differentiation of MSCs, the effect of their activity on changes in the epigenetic landscape remains elusive [68–70]. However, it becomes clear that HDAC4-6 expression is controlled by lncRNA H19 activity and lncRNA H19-derived miR-675 activity, which are downregulated during adipogenesis [71].

The differentiation process leads to the maturation of adipocytes and results in acquiring morphological and phenotypical characteristics as well as gene expression profiles of mature adipocytes. Importantly, once the adipogenic program is activated, MSCs lose their ability to differentiate towards other lineage cells and, therefore, are referred to as preadipocytes. Likewise, osteogenic differentiation inducers associated with the Wnt signaling pathway repress *PPARG* expression and, in consequence, block adipogenic differentiation [72]. The canonical Wnt/β-catenin pathway directly inhibits the expression of the *PPARG* gene. In contrast, the noncanonical Wnt pathway members such as Wnt5a promote adipogenesis at the initial stage of differentiation [73].

### Osteogenic differentiation

**Fig. 3:**
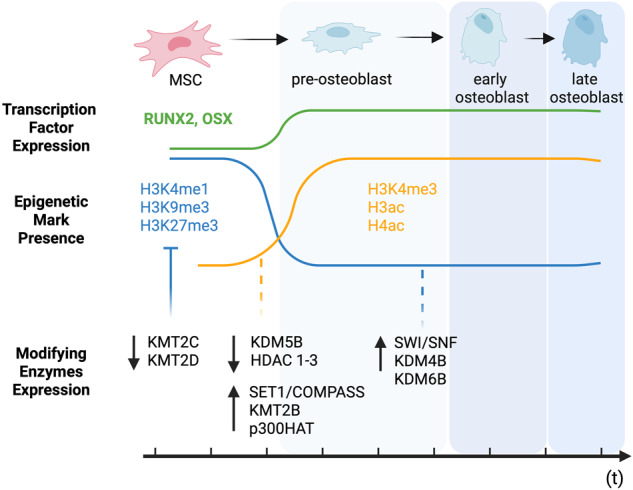
Epigenetic changes during osteogenic differentiation. Osteogenesis is a three-step process consisting of the proliferative phase, matrix maturation, and mineralization. The process is controlled by RUNX2 and OSX transcription factors. In MSCs, their expression is inhibited by repressive marks, namely histone H3 mono-methylation at the 4^th^ lysine residue (H3K4me1), histone H3 tri-methylation at the 9^th^ lysine residue (H3K9me3), histone H3 tri-methylation of 27^th^ lysine residue (H3K27me3), and DNA methylation at the 5^th^ carbon of cytosine (5mCpG). After induction of differentiation, expression of methyltransferases (KMT2C and KMT2D) is reduced, and repressive marks are removed by SWItch/Sucrose non-fermentable complex (SWI/SNF) and histone demethylases (KDM4B and KDM6B) activity. They are replaced by activation marks, namely histone H3 tri-methylation at the 4^th^ lysine residue (H3K4me3) and acetylation (ac) of histones H3 and H4, which opened the chromatin for RUNX2 and OSX expression. Histone acetylation is mediated by p300HAT (p300 histone acetyltransferase) and accompanied by decreased expression of HDAC1-3 (Histone deacetylases 1-3), while H3K4me3 is mediated by SET1/COMPASS and KMT2B activity and accompanied by reduced expression of KDM5B. Created with BioRender.com.

In contrast to adipogenesis, osteogenic differentiation is characterized as a three-step process starting with the proliferative phase, then matrix maturation, and mineralization (Fig. 3). Runt-related transcription factor 2 (RUNX2, also known as a core-binding factor-α-CBFA1) and Osterix (OSX; also known as Sp7) represent the main transcription factors for osteogenic differentiation [74–76]. Their expression is accompanied by increased miR-27b, miR-130a, activity that directly targets PPARγ expression [77]. Both transcription factors control the expression of numerous downstream genes encoding proteins crucial for cellular plasticity induction and osteoblast phenotype establishment. Activation of RUNX2 induces transformation into osteoblast-lineage cells by increasing the expression of hedgehog (*Indian Hedgehog Signaling Molecule (Ihh)*, *Gli Family Zinc Finger 1 (Gli1)*, and *Patched 1 (Ptch1)*), fibroblast growth factor (Fgf; *Fgfr1, Fgfr2*, and *Fgfr3*), Wnt family member (*Transcription Factor 7 (Tcf7)*, *Wnt1* and *Wnt10b*), and parathyroid hormone-like hormone (Pthlh; *Parathyroid Hormone 1 Receptor (Pthr1)*) signaling genes [78]. In addition, RUNX2 modulates the expression of bone-related genes, including osteocalcin (OCN), collagen I (COL1a1), osteopontin (OPN), bone sialoprotein (BSP), alkaline phosphatase (ALP), and the parathyroid receptor (PTHR) [79, 80]. However, both RUNX2 and OSX with simultaneous canonical Wnt and BMP signaling pathway activation are required for mature osteoblast phenotype establishment and their proliferation [81, 82].

Notably, transcription of both mentioned orchestrators of osteogenic differentiation undergoes epigenetic control. To date, described mechanisms are associated with changes in i) methylation at histone H3 residues; ii) acetylation at histone H3 and H4 residues; and iii) DNA methylation signatures [83, 84]. All the mechanisms attract both promoter regions of the described transcription factors. Repressive marks, which are associated mainly with histone H3 modifications (such as mono-methylation at the 4th lysine residue (H3K4me1 mark), tri-methylation of 9th lysine residue (H3K9me3 mark), and tri-methylation of 27th lysine residue (H3K27me3 mark)) and direct DNA methylation at the 5th carbon of cytosine (5mCpG), are reduced and replaced with active marks such as trimethylation of 4th lysine on histone H3 (H3K4me3 mark) and acetylation of histone H3 (H3ac) and H4 (H4ac) [85]. Osteogenic differentiation decreases the activity of histone-H3K4-specific demethylase KDM5B and consequently increases the H3K4me3 mark activating Runx2 transcription [86, 87]. This process seems to be associated with increased activity of lncRNA TCONS_00041960, TCONS_00023297, and CALB2 acting as a sponge for Runx2 targeting miR-204-5p, miR-608-5p, and miR-30b-3p respectively [88–90]. On the other hand, H3K4me3 mark enrichment in both promoters of described transcription factors may be mediated by histone-H3K4-specific methylases belonging to the Complex Proteins Associated with Set1 (COMPASS) family complexes, namely SET1/COMPASS and MLL2/COMPASS-like (also known as KMT2B) [91–93]. In contrast, MLL3/COMPASS-like (also known as KMT2C) and MLL4/COMPASS-like (also referred to as KMT2D) seem to be responsible for the maintenance of repressive marks and are downregulated after induction of osteogenic differentiation by monomethylation of the 4th lysine of histone H3 (H3K4me1 mark) [94, 95]. In addition, H3K9me3 and H3K27me3 repressive marks are reduced by the activity of SWI/SNF (belonging as mentioned above to the ATP-dependent chromatin remodeling complexes family), histone demethylases (KDM4B and KDM6B, respectively) resulting in increased osteogenic potential [96–99]. Furthermore, histone H3 and H4 acetylation are associated with a reduced expression of HDAC1-3 activity and a simultaneous increase of histone acetyltransferase p300 (p300 HAT) activity, allowing effective acetylation in both promoter regions of RUNX2 and OSX [100, 101]. This process is also controlled by the lncRNA H19/miR-675 regulation of HDAC4-6 expression [67]. However, similarly to adipogenic differentiation, the role of HDAC4-6 in orchestrating the epigenetic landscape remains not fully elucidated. It has been proposed that inhibition of HDAC4-6 reduces acetylation of histone H4 at the endogenous OCN promoter induced by TGF−β1 [102].

The process of bone formation decreases with aging and during inflammatory bone diseases. High levels of IL-1β or TNF pro-inflammatory cytokines suppress osteogenesis by BMP/Smad signaling inhibition [103, 104]. However, Sirt1 activation increases MSC potential to osteogenic differentiation by modulation of NF-κβ transcription factor and RUNX2 upregulation [105]. Moreover, *HOXB7* increased expression influence *ON*, *OCN*, *BSP*, and *COL1A2* gene expression and further *RUNX2* [106]. Importantly, RUNX2 high expression during proliferation phase promotes, in turn, distal-less homeobox 5 (DLX5) and bone sialoprotein (BSP) transcription factors [107, 108]. Furthermore, Satb2 contributes to MSC osteogenic differentiation [109]. However, the maturation and mineralization phase seems to be promoted by OSX, the second major transcription factor during osteogenesis, which adjusts the expression of Ocn, Opn, and Osteonectin (On) mature osteoblast genes [110]. Moreover, *ALP* expression increases through Runx2 influence [111].

### Chondrogenic differentiation

**Fig. 4:**
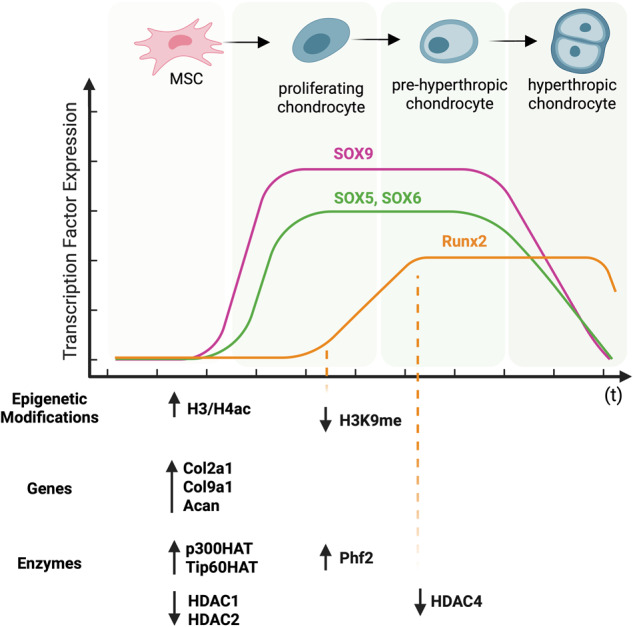
Epigenetic changes during chondrogenic differentiation. Chondrogenesis begins with mesenchymal condensation, followed by chondrocyte differentiation and maturation. SRY-Box Transcription Factor (SOX) 9, together with cofactors SOX5 and SOX6) represents a key transcription factor responsible for Collagen *(COL2a1, COL9a1,* and *COL11a1)* and *Aggrecan* (*Acan*) gene expression. The maturation phase is controlled by Histone H3 and H4 acetylation (ac) by p300HAT (p300 histone acetyltransferase) and Tip60HAT (Tip60 histone acetyltransferase). Chondrocyte maturation and hypertrophy are controlled by Runx2 expression. Its expression is associated with the demethylation of the 9^th^ lysine (K9) at histone H3 (H3K9me mark) by Phf2 (PHD Finger Protein 2) demethylase and histone deacetylase 4 (HDAC4) downregulation. Created with BioRender.com.

MSCs differentiation toward chondrocytes strictly depends on cellular density (condensation of cells into 3D structures that occurs with the central role of N-cadherin) and growth factors stimulation (tumor growth factor beta (TGFβ) family members are well established in this process, namely TGFβ1, TGFβ2, and TGFβ3) (Fig. 4) [112, 113].

Chondrogenesis commences with mesenchymal condensations (morphogenetic transformation mediated by cell adhesion), chondrocyte differentiation, and maturation. SRY-box containing gene 9 (*SOX9*) is a key transcription factor in early chondrogenesis [114]. Together with its cofactors, namely SRY-box containing gene 5 (SOX5) and SRY-box containing gene 6 (SOX6), control expression of cartilage-specific extracellular matrix genes (such as *COL2A1*, *COL9A1*, *COL11A2*, and *Aggrecan (ACAN))* [115]*. SOX9* expression is directly regulated by miR-145 and miR-495, and indirectly by miR-574-3p inhibiting retinoid x receptor alpha (RXRα) an inhibitor of chondrogenesis [116–119]. Moreover, Sox9 interacts with a number of differentiation stage-specific transcriptional regulators, such as WW domain-containing E3 Ubiquitin Protein Ligase 2 (Wwp2) transport to the cell nucleus. Wwp2 as a co-factor leads to increased expression of Sox9 [120]. Additionally, the transcription factor Zinc finger protein (Znf)219, associated by the C-terminal region with Sox9 factor activity on the *Col2a1* promoter, upregulate Sox9 expression during chondrocyte differentiation and lead to increased expression of *Col2a1, Col11a2*, and *ACAN* [121]. AT-rich interaction domain (Arid)5a, as another transcriptional regulator, stimulates chondrogenesis by binding directly to the *Col2a1* gene promoter [122]. Importantly, these activities are accompanied by changes in the epigenetic landscape involving both DNA methylation and histone modifications. In fact, chondrocyte-specific gene promoter regions (such as COL10A1) are hypomethylated in the course of MSC differentiation [123, 124]. In addition, histone acetylation has been shown to support chondrocyte differentiation [125, 126]. p300 HAT supports Sox9-mediated expression of cartilage-specific genes such as *Col2a1*, a major component of cartilage surrounding extracellular matrix, by acetylation of histones H3 and H4 in the enhancer regions [127]. Similarly, Tip60 HAT interacts with Sox9 and enhances its transcriptional activity in chondrocyte differentiation by the acetylation mark of lysine 61, 253, and 398 residues [128].

On the other hand, HDAC1- and HDAC2-mediated histone deacetylation significantly lowers the expression of extracellular matrix genes, including *Col2a1, Col9a1, and Acan*, as well as cartilage oligomeric matrix protein (a non-collagenous cartilage matrix protein) [129–131]. HDAC4 has been shown to inhibit *Runx2* expression, thus significantly reducing chondrocyte differentiation and hypertrophy [132]. In addition, nicotinamide adenine dinucleotide (NAD)-dependent deacetylase Sirtuin1 has been reported to reduce the expression of the *COL2A1* gene [133].

Unfortunately, our understanding of histone methylation in this process remains elusive. Similarly, our understanding of the role of ncRNAs in the regulation of epigenetic machinery during MSCs chondrogenic differentiation needs significantly more attention in the future. Some reports indicate an important role of H3K4me3 and H3K36me3 active marks [134, 135]. Moreover, H3K9 methylation has been shown to inhibit chondrocyte maturation and hypertrophy through the downregulation of *Runx2* expression. This repression mark may be erased by the involvement of AT-rich interactive domain 5b (Arid5b) recruited histone demethylase Phf2 to promote chondrogenesis [136].

## Epigenetic control of mesenchymal stem cells immunomodulatory properties

**Fig. 5:**
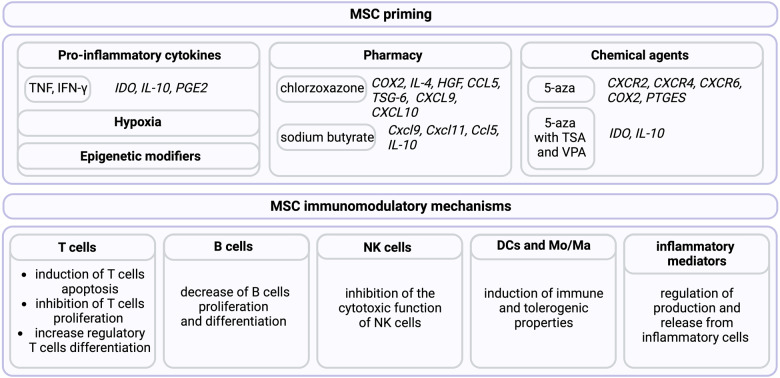
Summary of mesenchymal stem cell priming strategies for the induction of immunomodulatory activities. TNF – tumor necrosis factor; IFN – interferon; IL – interleukin; IDO - indoleamine 2,3-dioxygenase; PGE2 – prostaglandin E2; COX2 – cyclooxygenase 2; HGF - hepatocyte growth factor; CCL5 – CC chemokine superfamily member; CXCL – CXC chemokine superfamily member; TSG-6 - Tumor necrosis factor-inducible gene 6 protein; 5-aza – 5-Aza-2′-deoxycytidine; PTGES - prostaglandin E synthase; TSA - trichostatin A, VPA - valproic acid. Created with BioRender.com.

MSCs acquire immunosuppressive functions in a proinflammatory microenvironment (in response to cytokine stimulation such as IL-1α/β, IFNγ, TNF, and IL-17) [137, 138], hypoxia conditions [139], or in response to pharmacological drugs (bortezomib, dexamethasone) [140, 141] and chemical/biological agents (LL-37, LPS, curcumin, α-synuclein) [142–145], including epigenetic modifiers (HDAC/DNMT inhibitors) [146, 147]. To date, several MSCs mediated immunomodulatory mechanisms have been described, including (1) reduction of T cell-mediated responses by induction of T cell apoptosis, inhibition of T cell proliferation, and supporting of regulatory T cell differentiation [148]; (2) limitation of B cell responses by regulation B cell proliferation and differentiation towards plasma cells [149]; (3) inhibition of the cytotoxic function of natural killer (NK) cells [150]; (4) induction of dendritic cells (DCs) [151] and monocyte/macrophage (Mo/Ma) tolerogenic properties [152]; and (5) limitation of inflammatory mediator secretion (Fig. 5) [153].

The MSCs stimulation with pro-inflammatory cytokines leads to an increase in indoleamine 2,3-dioxygenase (IDO) levels, high IL-10 and prostaglandin E2 (PGE2) secretion, and induction of immunoinhibitory checkpoint (coinhibitory) molecule expression; all of these activities are recognized as hallmarks of their immunomodulatory functions [154, 155]. Interestingly, increased immunosuppressive activities have been observed after MSC priming with a combination of proinflammatory IFNγ and polyinosinic-polycytidylic acid (poly(I:C), a synthetic analog of double-stranded RNA, a ligand for toll-like receptor 3 (TLR3) stimulation [156–158]. However, it remains elusive how epigenetic machinery is involved in the proinflammatory cytokine-mediated MSCs licensing. In fact, our understanding of epigenetic mechanisms that control immunosuppressive functions of MSCs comes mainly from the reports describing the in vitro effect of chemical stimulations, including hypomethylating agents (5-aza-2’-deoxycytidine) (5-aza), HDAC inhibitors (trichostatin A and valproic acid), and drugs known to act on epigenetic marks (chlorzoxazone, sodium butyrate) [159–162].

5-aza is a chemical analog of cytidine, which possess hypomethylating activity and direct cytotoxicity in higher doses. In clinical practice, it is used to treat some types of malignancies, such as myelodysplastic syndrome (MDS) and acute myeloid leukemia (AML) [163]. In MSCs, 5-aza-2’-deoxycytidine has been shown to alter their differentiation potential and gene expression profiles, including enhancement of immunomodulatory gene expression. It leads to the demethylation of *CXCR2, CXCR4, and CXCR6* promoters, which enhances MSCs migration potential in response to IL-8, C-X-C Motif Chemokine Ligand (CXCL1), and SDF-1 gradient [164]. The combination treatment of 5-aza with histone deacetylase inhibitors (trichostatin A and valproic acid-selectively inhibiting class I and II histone deacetylases) resulted in elevated expression of *IDO* and *IL10*. These licensed MSCs exerted a regulatory effect on Th1 and Th17 cell differentiation and significantly decreased IFNγ, IL-2, and IL-17 levels [165]. Microarray and methylation analyses revealed the demethylation of 7734 gene promoters and methylation of 5615 genes induced by 5-aza treatment of MSCs in inflammatory conditions. The pretreatment with 5-aza decreases methylation of immunomodulatory promoters *COX2 and PTGES*, and migration factors *CXCR2* and *CXCR4*, subsequently contributing to the increased PGE_2_ production by MSC and suppress lymphocyte proliferation [147].

Deng L. et al. recently proposed chlorzoxazone for improving MSCs immunosuppressive properties in rat acute kidney injury model. Chlorzoxazone is a benzoxazole derivative used in clinical practice as a muscle-relaxing drug. Chlorzoxazone, in contrast to IFNγ stimulation, does not induce changes in the biological characteristic of MSCs and their MHC I and MHC II expression while similarly inhibiting the T cell activation and proliferation *via* the increase in IDO expression [160]. Moreover, chlorzoxazone elevates the expression of *Cyclooxygenase (COX2)*, *IL-4*, *Hepatocyte Growth Factor (HGF)*, *TNF-stimulated gene 6 (TSG-6)*, *C-C Motif Chemokine Ligand 5 (CCL5; RANTES)*, *C-X-C Motif Chemokine Ligand (CXCL9*, and *CXCL10 (IP-10))* [160]. At the epigenetic level, chlorzoxazone has been shown to regulate the phosphorylation of transcription factor Forkhead Box O3 (FOXO3) independently to AKT and ERK signaling pathways [160]. Administration of chlorzoxazone-educated MSCs resulted in the reduction of renal tissue infiltration with immune cells and impairment of fibrinoid necrosis within the glomeruli, compared to naïve MSCs [160]. In addition, Yu T. et al. showed that inhibition of Tet-1 and Tet-2 demethylases in periodontium-derived MSCs (periodontal ligament stem cells) enhances their immunosuppressive functions. Tet-1 and Tet-2 knockdown results in *DKK1* promoter hypermethylation and a decrease of *DKK1* expression, leading to the activation of the Wnt/b-catenin signaling pathway and modulation of Fas ligand (FasL) expression. Consequently, MSCs acquire the ability to induce T cell apoptosis and increase the frequency of Tregs [166].

Similarly, to the above-mentioned drugs, stimulation of MSCs with IFNγ causes an increase in IDO enzyme level, resulting in the accumulation of toxic tryptophan metabolites that in turn, hamper T cell proliferation [167]. The expression of *Ido1* gene seems to be associated with a combination of hypomethylating agents and histone deacetylation inhibitors (HDACIs) [168]. The increase in H3K9ac is accompanied simultaneously by a reduction in H3K9me3 at the promoter region [169].

Taking together, differentially sourced MSCs may vary in their immunosuppressive properties, and distinct strategies for empowering therapy should be assessed with a focus on the changes in biological characteristics, immune privilege, and functionality of MSCs [170–172]. Therefore, there is a substantial need to strengthen our understanding of the mechanisms and immunomodulatory potential of MSCs isolated from different tissues [165, 166, 173, 174]. This certainly would bring us closer to their use in clinical practice.

## Perspective for improving clinical strategies involving MSCs

The approaches utilizing infusion or transfer of MSCs into patients have begun to develop in the 90’s of the last century. Over the past decades, the advances in MSCs administration procedures and safety strategies allowed for registering more than 950 clinical trials involving ~10,000 treated patients [175]. The considerable interest of the scientific community in optimizing MSC-based therapies in inflammatory and degenerative diseases resulted in the registration of more than 1000 clinical trials with a targeted enrollment of almost 50,000 patients in 2011–2018 [175]. Unfortunately, this great focus on clinical settings did not provide potent progress in the introduction of the first MSC-based therapy for routine medical practice.

Therefore, further efforts should be focused on enhancing the stability and effectiveness of MSCs transfer regardless of various conditions affecting the replicability of individual transplants. Given the complexity and diversity of the isolation procedures and expansion methods of MSCs, along with donor diversity and the heterogeneity in the inflammatory profiles of the recipient microenvironment, the achievement of the desired therapeutic outcome is very difficult. The advancement of two strategies might be useful in order to minimize currently defined issues, namely: (1) optimization of the MSC-related effects in controlled in vitro production conditions containing the standardized procedures using effective stabilizing/enhancing agents (such as inflammatory mediators, epigenetic or genetic modifiers); or (2) prediction suited properties and modification the MSC for personalized clinical indication (extensively reviewed in ref. [175]).

Recently developed methods, including single-cell sequencing (with multi-omics approaches) and further exploring of next-generation technologies, may progress our understanding of MSCs heterogeneity being the result of donors’ diversity, culturing conditions (hypoxia, length of culture), and isolation tissue sources [176]. Those advances may help to predict particular epigenetic manipulations or gene silencing, which might improve the desired therapeutic effect in individual clinical indications. On the other hand, the acquirement of a sufficient number of MSCs (hundreds of millions) for transplantation requires extensive expansion of cells in a standardized laboratory environment. The senescence of cells during culturing remains one of the leading problems in the advancement of cellular therapies. Aging MSCs may manifest loss of stemness, differentiation abilities, and immunomodulatory potential (for review please see ref. [177]). Therefore, epigenetic manipulation may maintain their beneficial properties or reverse the effects of senescence.

The improvement and substantial progress in the development of stem cell-based therapies require extensive research in the field of epigenetic machinery driving immunomodulatory activities and the differentiation potential of MSC. It is tempting to speculate that a better understanding of the role of epigenetic machinery and dynamic changes of the epigenetic landscape during fate decision, differentiation, and induction of immunomodulatory activities will allow us to better control the stability and enhance the therapeutic potential of MSCs.
